# Added effects of dual-task water-based exercise on global cognition and physical performance in older women: a three-arm randomized controlled trial

**DOI:** 10.3389/fphys.2026.1866915

**Published:** 2026-09-25

**Authors:** Camila Miranda, Lúcia Faria Borges, Andressa Martins e Silva, Katholyn Penna de Bitencourt, Mariana de Souza Strehl, Juliana Loesch, Cristine Lima Alberton, Andréa Kruger Gonçalves, Aline Nogueira Haas, Ana Carolina Kanitz

**Affiliations:** 1 School of Physical Education, Physiotherapy and Dance, Postgraduate Program in Human Movement Sciences, Federal University of Rio Grande do Sul, Porto Alegre, Brazil; 2 School of Physical Education and Physiotherapy, Postgraduate Program in Physical Education, Federal University of Pelotas, Pelotas, Brazil; 3 Global Brain Health Institute, Trinity College Dublin, Dublin, Ireland

**Keywords:** aquatic exercise, dual-task training, executive function, functional mobility, Montreal Cognitive Assessment (MoCA), physical fitness

## Abstract

**Introduction:**

Dual-task water-based exercise has been proposed to enhance attention and executive functions and yield cognitive benefits beyond conventional aquatic aerobics exercise. However, evidence remains limited and heterogeneous, as few randomized controlled trials have directly compared dual-task aquatic interventions with conventional aquatic program of equivalent dose in older women.

**Methods:**

This three-arm, parallel-group, superiority randomized controlled trial was conducted in Porto Alegre, Brazil, at ESEFID/UFRGS. Older women were allocated to: dual-task water-based exercise (WEDT), conventional water-based exercise (WE), or a non-exercise control group (CG). The intervention lasted 14 weeks (2 sessions/week). Global cognition (MoCA total score) was the primary outcome. Secondary outcomes included specific cognitive measures, mobility (Timed Up and Go [TUG] and dual-task TUG [TUG-DT]), aerobic capacity, muscular performance, maximal voluntary isometric contraction (MVIC), and rectus femoris and vastus lateralis electromyographic activity (EMG). Outcomes were assessed at baseline and post-intervention. Intention-to-treat analyses used generalized estimating equations (GEE) with Bonferroni *post hoc* tests; per-protocol analyses (≥70% attendance) were conducted as sensitivity analyses.

**Results:**

Of 186 individuals screened, 67 were randomized (WEDT n=23; WE n=23; CG n=21). Mean attendance in ITT was 55.2% ± 10.36% (WEDT) and 65.7% ± 9.89% (WE), and no intervention-related adverse events were reported. In ITT, MoCA total score showed no main effects of time or group and no group×time interaction (p>0.05); among MoCA domains, only orientation showed a main effect of time (p=0.006). Semantic verbal fluency showed a significant group×time interaction (p=0.003), increasing in WEDT, decreasing in WE, and remaining stable in CG. TUG showed no significant effects (p>0.05), whereas TUG-DT showed a significant group×time interaction (p=0.032), driven by improvements in CG. A significant main effect of group was observed for the 6-min walk test (p=0.020). Neuromuscular outcomes showed significant group×time interactions for handgrip strength (p=0.037; increases in WE), MVIC (p=0.014), and rectus femoris activation (p=0.028).

**Conclusions:**

In older women, adding periodized dual-task demands to aquatic aerobics did not provide consistent incremental benefits for global cognition, while physical effects were modest and heterogeneous. These findings should be interpreted considering the moderate adherence and reduced intervention dose actually received; future trials should evaluate higher training doses and strategies to enhance adherence and monitoring.

**Trial registration:**

RBR-4tzrdjz.

## 1 Introduction

Aging is a heterogeneous process and is often accompanied by cognitive and physical changes that vary in magnitude across individuals (Jost and Kujach, 2025). These alterations have been linked to age-sensitive brain structures, such as the prefrontal cortex, hippocampus, and temporal lobe, including reductions in volume and perfusion, with downstream effects on functions such as memory and planning (Domingos et al., 2021). Declines in attention and executive functions may also compromise physical performance, reduce independence, and hinder everyday activities frequently performed under dual-task conditions (Pettigrew and Soldan, 2019). These issues are particularly relevant in older women, who comprise the majority of the long-lived population and show a higher prevalence of functional limitations than men (Bloomberg et al., 2021; Farrelly, 2023).

Several strategies have been adopted to preserve cognitive function in older adults, notably pharmacological treatments and structured physical exercise (Wan et al., 2024). However, pharmacological interventions may be associated with adverse effects and have limited effectiveness in slowing the progression of dementias (Petersen et al., 2018). In contrast, regular physical exercise has been linked to neuroprotective effects, particularly by promoting mechanisms related to neuroplasticity, that is, the brain’s capacity to reorganize its structure and function in response to stimuli (Mansoor et al., 2025). In addition, exercise can improve physical and functional performance outcomes, further enhancing the clinical relevance of this type of intervention (Niyazi et al., 2024).

Water-based exercise, such as aquatic aerobics, represents an attractive option for older adults because it can improve physical performance outcomes, including mobility, balance, and cardiorespiratory fitness (Costa et al., 2018; Andrade et al., 2020; Häfele et al., 2023; Buitrago-Restrepo et al., 2024; Andrade et al., 2026), and may also benefit cognitive outcomes (Fedor et al., 2015; Kang et al., 2020; Farinha et al., 2021; Campbell et al., 2023). Water-based exercise offers unique advantages compared with land-based exercise modalities due to the physical properties of water. Buoyancy reduces body weight loading on joints, facilitating movement execution and reducing musculoskeletal stress (Alberton et al., 2019; Alberton et al., 2020), while hydrostatic pressure may improve venous return and cardiovascular responses (Pendergast et al., 2015). In addition, water viscosity provides continuous multidirectional resistance, allowing simultaneous aerobic and neuromuscular stimulation with lower impact and perceived risk of injury (Torres-Ronda and Del Alcázar, 2014). These characteristics may be particularly beneficial for older adults, who frequently present joint discomfort, balance impairments, fear of falling, and reduced exercise tolerance (Buitrago-Restrepo et al., 2024). Consequently, aquatic exercise represents an attractive and accessible strategy to promote both physical and cognitive health in aging populations (Costa et al., 2018; Buitrago-Restrepo et al., 2024; Andrade et al., 2026). However, cognitive findings from conventional aquatic protocols are not consistent. Reviews have reported positive effects on neuropsychological outcomes, including global cognition (Campos et al., 2021), whereas programs without specific cognitive stimulation do not always show cognitive improvement (Farinha et al., 2021; Häfele et al., 2023).

In this context, incorporating cognitive tasks into training in a dual-task format has been proposed as a strategy to engage attention and executive functions and to better reflect everyday demands (Campos-Magdaleno et al., 2022). From a theoretical perspective, combining physical exercise with concurrent cognitive challenges may provide additional benefits by simultaneously stimulating motor and cognitive neural networks, thereby increasing cognitive engagement and promoting neuroplastic adaptations (Lauenroth et al., 2016). This rationale is particularly relevant for older adults, as executive functions, attention, and dual-task performance are essential for maintaining independence in daily activities (Wollesen and Voelcker-Rehage, 2014). Supporting the potential relevance of the aquatic environment for dual-tasking, an acute study reported fewer errors during dual-task performance under water immersion compared with a land-based condition (Schaefer et al., 2016). Although recent evidence suggests potential benefits of combining aquatic exercise with cognitive stimulation, available findings still come from a small number of studies using heterogeneous designs, including pilot work and randomized trials that do not directly compare dual-task protocols with an equivalent conventional aquatic program (Sato et al., 2015; Campbell et al., 2023; Dunlap et al., 2024; Terra de Oliveira et al., 2024). Moreover, positive results have been reported mainly in individuals with mild cognitive impairment (Meekum et al., 2025).

Accordingly, it remains unclear whether adding a dual-task component to water-based exercise yields incremental benefits for cognition compared with conventional aquatic exercise, and whether increasing cognitive–motor demands can be achieved without compromising physical performance. Evidence from trials with this design is needed to better inform the prescription of aquatic exercise programs in practice. Therefore, the aim of this study was to investigate the additional effects of dual-task training within an aquatic aerobics program, compared with a conventional aquatic aerobics program and a non-exercise control group, on global cognition (primary outcome) in older women. Secondary outcomes included executive function, mobility, and physical performance. We hypothesized that the dual-task aquatic aerobics group would show greater improvements in global cognition and other cognitive outcomes than the other groups. We also expected that both exercise groups would improve physical performance relative to the control group, with larger gains in the conventional aquatic aerobics group, consistent with a potential cognitive–motor trade-off when cognitive demands are incorporated concurrently into training.

## 2 Materials and methods

### 2.1 Study design

This was a three-arm, parallel-group randomized controlled trial, conducted in accordance with CONSORT recommendations (Hopewell et al., 2025) and the Declaration of Helsinki. The trial was conducted in Porto Alegre, Rio Grande do Sul, Brazil, and all assessments and water-based training sessions were carried out at the School of Physical Education, Physiotherapy and Dance of the Federal University of Rio Grande do Sul (ESEFID/UFRGS). Participants were allocated to: dual task water-based exercise group (WEDT), conventional water-based exercise group (WE), and a non-exercise control group (CG). Assessments were performed at baseline and one week after the end of the intervention by assessors blinded to group allocation. The study was approved by the Research Ethics Committee of the Federal University of Rio Grande do Sul (CAAE: 75529023.6.0000.5347) and registered in the Brazilian Registry of Clinical Trials (RBR-4tzrdjz).

### 2.2 Participants

The participants were recruited between June and July 2024, and follow-up assessments were completed in November 2024. Community-dwelling older adults were recruited if they met the following inclusion criteria: age ≥ 60 years; independent ambulation; a medical certificate authorizing participation in physical exercise; ability to understand verbal instructions required to complete the tests; availability to attend the training sessions; MoCA score ≥ 17; and no participation in structured exercise training programs in the three months preceding study entry. Exclusion criteria included clinical/neurological conditions and/or limitations that would preclude the safe execution of the intervention protocol or the assessments.

#### 2.2.1 Sample size

Sample size was estimated *a priori* using G*Power (version 3.1), considering the primary outcome (global cognition), a repeated-measures model with three groups and two time points (pre- and post-intervention), a 5% significance level (α = 0.05), and 80% statistical power (1−β = 0.80). Based on Farinha et al. (2021), we assumed an expected effect size of f = 0.25, yielding a minimum sample of 42 participants (14 per group). To account for potential losses/attrition, 30% was added, resulting in 54 participants eligible for randomization.

#### 2.2.2 Randomization, allocation concealment, and blinding

Eligible participants were randomized to the experimental groups or control using a stratified randomization procedure based on age, and baseline MoCA score. The random allocation sequence was generated by an independent researcher using a computer-generated random list (Microsoft Excel, RAND) function). This procedure was performed after completion of baseline assessments by a researcher who was blinded to baseline results interpretation and not involved in outcome assessments. After completion of baseline assessments, participants were assigned according to a sequentially numbered allocation list, which was accessed only after participant enrollment and completion of baseline testing. Participants were informed of their group assignment by the study coordinator. Outcome assessors remained blinded to group allocation throughout data collection.

### 2.3 Interventions

The interventions were delivered in a swimming pool measuring 16 m in length, 6 m in width, with a depth ranging from 1.40 to 1.60 m. Participants were instructed to maintain immersion at the level of the xiphoid process. Water temperature was maintained between 31 and 32 °C. Sessions were conducted in groups twice weekly on non-consecutive days and were supervised by professionals experienced in aquatic exercise. Before the training period, participants in both intervention groups completed four familiarization sessions with the aquatic environment, the exercises, and the Borg Rating of Perceived Exertion (RPE) scale (Fedor et al., 2015; Costa et al., 2018; Petersen et al., 2018; Alberton et al., 2019; Alberton et al., 2020; Andrade et al., 2020; Kang et al., 2020; Farinha et al., 2021; Campbell et al., 2023; Häfele et al., 2023; Buitrago-Restrepo et al., 2024; Niyazi et al., 2024; Wan et al., 2024; Mansoor et al., 2025; Andrade et al., 2026), which was used to prescribe exercise intensity.

#### 2.3.1 Conventional water-based exercise

Sessions lasted 45 minutes and comprised a warm-up (5 min), main phase (30 min), and cool-down (10 min). The main phase consisted of an aerobic-focused aquatic exercise (aquatic aerobics) program with progressive increases in intensity across the weeks. Exercise intensity was prescribed using the Borg RPE scale, with a planned progression from 13 to 17 (moderate to very hard) according to the training period. However, individual RPE values were not systematically recorded during or after the sessions. The WE group performed a conventional water-based program consisting of general upper- and lower-limb movements (e.g. stationary running, sky cross country, and arm exercises performed against water resistance) aimed at improving cardiorespiratory fitness. A detailed description of the sessions, including examples of activities performed and intensity progression across training blocks, is provided in the **Supplementary Material** (**Supplementary Tables S1**).

#### 2.3.2 Dual-task water-based exercise

The WEDT group completed the same protocol as the WE group (i.e., identical session structure and intensity progression), with the inclusion of 10 minutes of cognitive stimuli in a dual-task format during the main phase. For this purpose, the 30-minute main phase was structured into 10 sets of 3 minutes each. Each set consisted of 2 minutes of exclusively motor activities followed by 1 minute of combined cognitive-motor activity, resulting in a total of 10 minutes of dual-task exercise. The cognitive component was designed to increase cognitive demand throughout the program. To guide this progression, tasks were structured based on the domains assessed by the Montreal Cognitive Assessment (MoCA), with gradual increases in complexity and/or attentional load over the weeks. Progression involved increments in task difficulty while maintaining simultaneous performance with the motor task. A detailed description of the tasks, targeted cognitive domains, and the periodized progression is provided in the **Supplementary Material** (**Supplementary Table S2**) to support reproducibility and implementation in other contexts.

#### 2.3.3 Safety monitoring

Adverse events were defined as any injury, symptom, or health complaint occurring during or immediately after the exercise sessions and considered potentially related to the intervention. Participants were asked about possible intervention-related adverse events during the supervised sessions, and any reported event was documented using a standardized adverse event form. Health problems unrelated to the intervention were recorded separately as reasons for absence or withdrawal.

#### 2.3.4 Control group

The participants in the control group were instructed to maintain their usual routines and refrain from initiating any structured exercise program during the study period. No additional recommendations regarding cognitive tasks or cognitive training were provided.

### 2.4 Outcomes and assessment procedures

The primary outcome was global cognition, assessed by the MoCA total score at baseline and 1-week post-intervention. Effects were evaluated using the change over time between groups (group×time interaction; i.e., differential pre–post change). Secondary outcomes were assessed at the same time points and included phonemic and semantic verbal fluency, Trail Making Test Parts A and B (completion time), functional mobility (Timed-up and go -TUG- and dual-task TUG completion time), aerobic capacity (6-minute walk distance), lower-limb muscular endurance (30-s sit-to-stand repetitions), handgrip strength, maximal isometric knee extensor strength, and rectus femoris and vastus lateralis electromyographic activity.

All assessments were conducted at the ESEFID/UFRGS Olympic Center and were distributed across two visits. During the first visit, participants completed a health history interview, followed by anthropometric measurements, cognitive assessments, and physical performance tests. During the second visit, maximal isometric knee extension strength and electromyographic activity of the rectus femoris and vastus lateralis muscles were evaluated. The order of the physical performance tests during the first visit was as follows: Timed Up and Go (TUG), dual-task TUG (TUG-DT), 30-s sit-to-stand test, handgrip strength, and 6-min walk test.

#### 2.4.1 Cognitive assessment

Global cognition (primary outcome) was assessed using the MoCA (score range 0–30), a widely used screening tool for cognitive impairment in older adults (Nasreddine et al., 2005). As secondary cognitive outcomes, the MoCA domains were also analyzed (visuospatial/executive, naming, attention, language, abstraction, memory, delayed recall, and orientation). Verbal fluency was assessed using phonemic (words beginning with a predetermined letter) and semantic (category) tasks, with the number of valid items produced within the allotted time recorded (Brucki et al., 1997). Executive functions and attention were assessed using the Trail Making Test Parts A and B, with completion time and errors recorded from a single administration according to standardized procedures (Magila and Caramelli, 2000).

#### 2.4.2 Functional mobility: TUG and dual-task TUG

Functional mobility was assessed using the TUG test and the dual-task TUG (TUG-DT). In the TUG, participants rose from a chair, walked 3 m, turned, returned to the chair, and sat down, with total time recorded. In the TUG-DT, the same course was completed while performing a concurrent cognitive task consisting of serial subtraction (counting backwards) from a randomly selected number between 70 and 100, with total time recorded (Podsiadlo and Richardson, 1991). Two trials were performed for each test, and the best performance was used for analysis.

#### 2.4.3 Aerobic capacity: 6-min walk test

Aerobic capacity was estimated using the 6-min walk test, following standardized procedures, with the total distance covered on a flat surface recorded (Rikli and Jones, 1999). One trial was performed.

#### 2.4.4 Lower-limb muscular endurance: 30-s sit-to-stand test

Lower-limb muscular endurance was assessed using the 30-s sit-to-stand test. The number of full repetitions completed within the allotted time was recorded, according to the described protocol (Rikli and Jones, 1999). One trial was performed.

#### 2.4.5 Handgrip strength

Handgrip strength was measured using a hand-held dynamometer (JAMAR, Bolingbrook, USA). Participants performed the test in a seated position and were instructed to exert maximal grip force with the dominant hand, with the dominant upper limb supported on the chair armrest. Three maximal trials were performed with 1-min rest intervals, and the highest value was retained for analysis (Hamilton et al., 1992).

#### 2.4.6 Maximal isometric knee extensor contraction and electromyography

Maximal isometric knee extensor strength was assessed using a maximal voluntary isometric contraction (MVIC) of the dominant leg. Participants were positioned on a knee extension chair with the hip at 90°flexion and the knee at 60°flexion; joint angles were measured using a goniometer. Participants were instructed to produce maximal force as quickly as possible. Three trials were performed, each lasting 5 s, with 2-min rest intervals between trials. Throughout all trials, researchers provided standardized verbal encouragement to ensure maximal effort. Force output was recorded using a load cell connected to an analog-to-digital converter (MIOTEC, Porto Alegre, Brazil).

Aligned with force acquisition from the load cell, neuromuscular activity of the rectus femoris (RF) and vastus lateralis (VL) muscles was assessed using surface electromyography. Electrodes were placed in a bipolar configuration (20-mm interelectrode distance) parallel to muscle fiber orientation, following the SENIAM (2024), guidelines (Nasreddine et al., 2005). Skin preparation, including shaving and gentle abrasion at the recording site, was performed to keep impedance below 3 kΩ. The reference electrode was placed over the patella. Raw electromyographic (EMG) signals were collected using an 8-channel system (New Miotool, MIOTEC, Porto Alegre, Brazil) at a sampling rate of 2000 Hz. To ensure consistent electrode placement between pre- and post-intervention assessments, anatomical landmarks and skin marks near the electrode sites were traced onto transparent sheets (Narici et al., 1989).

The highest and most stable force curve was selected for analysis. EMG signals from the RF and VL muscles were processed using a custom routine written in Python (v3.8). First, offset was removed from the raw EMG and force signals. Raw EMG signals were then band-pass filtered using a 4th-order Butterworth filter (20–450 Hz), and the force signal was low-pass filtered using a 4th-order Butterworth filter (6 Hz). Next, root mean square (RMS) values were computed from the EMG signals using 100-ms windows. The most **sTable 1-s** epoch with the highest force output during the test was visually identified based on the force curve. Finally, the mean and standard deviation over this 1-s epoch were calculated from the EMG RMS curves for the RF and VL muscles.

### 2.5 Adherence, monitoring, and safety

Attendance was recorded using sign-in sheets. Adverse events and intercurrent health issues were monitored throughout the intervention period. Session intensity was not systematically monitored (e.g., no session RPE values were recorded).

### 2.6 Statistical analysis

Continuous variables are presented as mean and standard deviation and 95% confidence intervals, whereas categorical variables are reported as absolute and relative frequencies. Normality and homogeneity of variances were assessed for baseline continuous variables using the Shapiro–Wilk and Levene tests, respectively. These procedures were used only for baseline analyses. For baseline comparisons of continuous sample-characterization variables across the three groups, a one-way analysis of variance (ANOVA) was used. For categorical variables, Pearson’s chi-square test was applied to examine potential differences in frequency distributions.

Generalized estimating equations (GEE) with Bonferroni *post hoc* tests were used to examine main effects of group and time, and the group × time interaction, for all outcomes. No baseline characteristics were included as covariates in the GEE models. The primary analysis followed the intention-to-treat principle with an available-case approach: all randomized participants were analyzed according to their original group allocation, and all observed outcome data were included in the models. Missing post-intervention values were not imputed; therefore, participants with incomplete follow-up contributed only the measurements available for the corresponding outcome. The GEE models accounted for within-participant correlations across repeated measurements. As a sensitivity analysis, a per-protocol (PP) analysis was conducted including only participants who completed both pre- and post-intervention assessments and achieved ≥ 70% adherence to the scheduled sessions (**Supplementary Material, Supplementary Table S3**). The statistical models and significance criteria used in the PP analysis were the same as those applied in the ITT analysis. Statistical analyses were performed using IBM SPSS^®^ software (version 23.0), with α = 0.05. Effect sizes (ES) were calculated using Hedges’ g based on post-intervention values, comparing each intervention group with the control group, and were classified according to Brydges (2019): small, ≤ 0.15; moderate, > 0.15 to ≤ 0.40; large, > 0.40 to ≤ 0.75; and very large, > 0.75.

## 3 Results

A total of 186 individuals expressed interest in participating in the study; 119 were excluded for not meeting eligibility criteria (age < 60 years, scheduling incompatibility, regular exercise participation, MoCA score < 17, and health conditions precluding participation), for refusing to sign the informed consent form, or due to loss to follow-up after initial contact, as shown in the study flow diagram (**Figure 1**). Consequently, 67 participants were randomized and included in the intention-to-treat analysis: 23 allocated to the WEDT group, 23 to the WE group, and 21 to the control group. In the intention-to-treat analysis, mean attendance was 55.2% ± 10.36% in the WEDT group and 65.7% ± 9.89% in the WE group, and no intervention-related adverse events were reported. For the per-protocol sensitivity analysis, 12 participants in the WEDT group, 14 in the WE group, and 16 in the control group were included, with mean attendance of 89.2% ± 8.9% and 82.9% ± 5.4% in the WEDT and WE groups, respectively.

**Figure 1:**
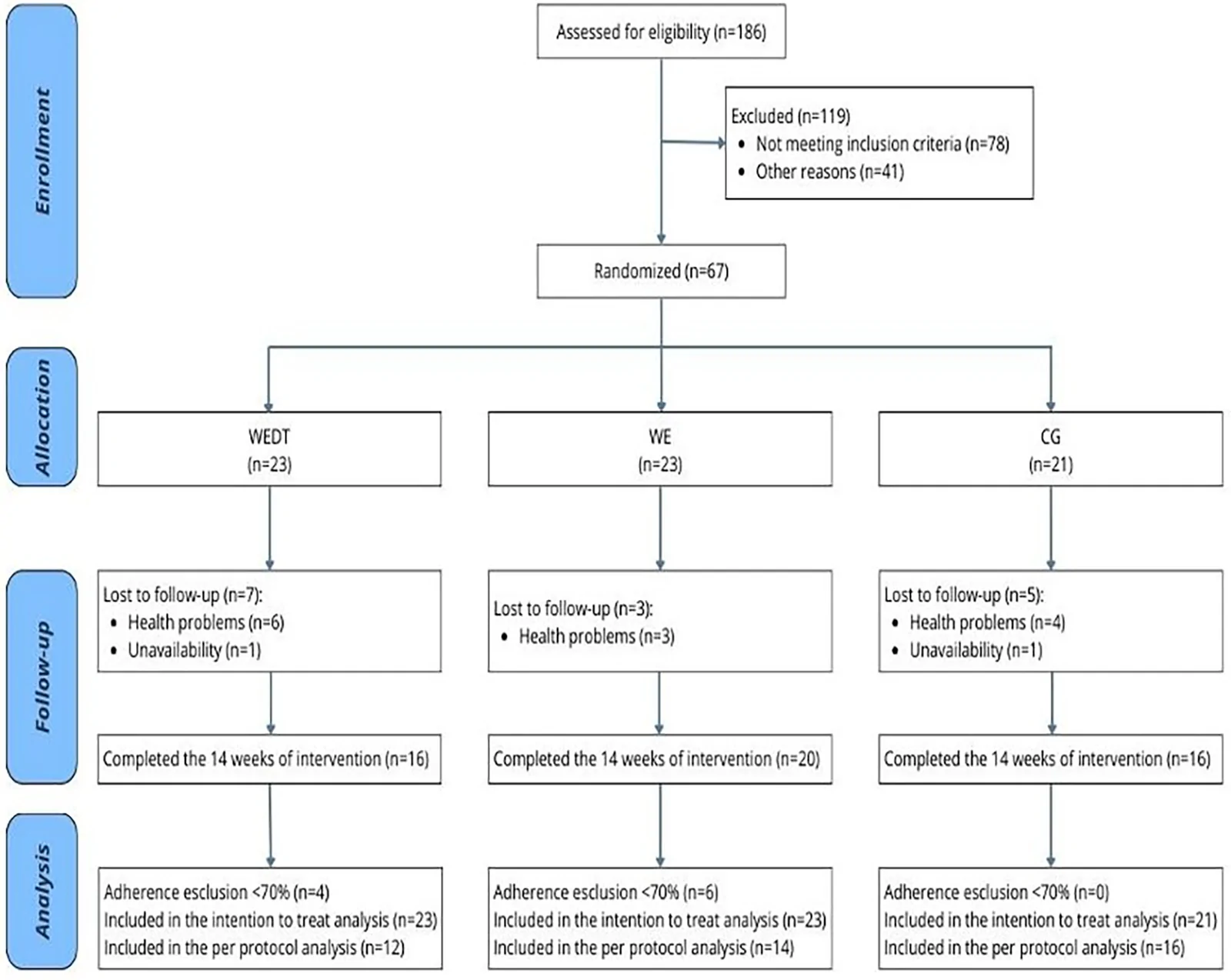
Participant flow through the study (CONSORT diagram).

Analysis of sample characteristics showed no between-group difference for most variables. Race was the only variable that differed, with a higher proportion of participants self-identifying as Black in WE group (**Table 1**).

**Table 1:** Baseline participants’ characteristics.

Variable	WEDT (n=23) Mean (SD)/ n (%)	WE (n=23) Mean (SD)/ n (%)	CG (n=21) Mean (SD)/ n (%)	*p*
Age (years)	69.81 ( ± 5.98)	69.00 (± 6.27)	67.14 ( ± 5.22)	0.359
Body Mass (kg)	80.35 ( ± 13.59)	74.74 (± 10.88)	76.07 ( ± 15.05)	0.396
Height (m)	1.60 ( ± 0.07)	1.59 ( ± 0.05)	1.61 ( ± 0.08)	0.690
BMI (kg/m²)	31.56 ( ± 5.51)	28.12 ( ± 7.49)	27.89 ( ± 7.71)	0.477
WHR (cm)	0.92( ± 0.06)	0.89 ( ± 0.22)	0.86 ( ± 0.10)	0.287
Race				
White	22 (95.6%)	14 (60.9%)	20 (95.2%)	0.042*
Mixed-race	0 (0%)	1 (4.3%)	0 (0%)	
Black	1 (4.4%)	8 (34.8%)	1 (4.8%)	
Marital Status				
Married	12 (52.2%)	10 (43.5%)	12 (57.2%)	0.735
Divorced	6 (26.1%)	5 (21.7%)	1 (4.8%)	
Single	1 (4.3%)	2 (8.7%)	4 (19.0%)	
Widowed	4 (17.4%)	6 (26.1%)	4 (19.0%)	
Education				
≥12 years of schooling	11 (47.8%)	9 (39.1%)	10 (47.6%)	0.615
8–11 years of schooling	10 (43.5%)	13 (56.5%)	8 (38.1%)	
4–7 years of schooling	1 (4.3%)	1 (4.4%)	3 (14.3%)	
Other	1 (4.3%)	0 (0%)	0 (0%)	
Monthly income				
≤ 1 SM	5 (21.7%)	5 (21.7%)	4 (19.0%)	0.059
1–3 SM	13 (56.5%)	16 (69.6%)	9 (43.0%)	
4–6 SM	5 (21.7%)	1 (4.3%)	4 (19.0%)	
7–9 SM	0 (0%)	1 (4.3%)	4 (19.0%)	
≥9 SM	0 (0%)	0 (0%)	0 (0%)	
Living arrangement				
Partner	9 (39.1%)	9 (39.1%)	12 (57.1%)	0.451
Child (ren)	4 (17.4%)	7 (30.4%)	1 (4.8%)	
Sibling(s)	0 (0%)	1 (4.3%)	1 (4.8%)	
Lives Alone	10 (43.5%)	5 (21.7%)	7 (33.3%)	
Grandchild (ren)	0 (0%)	1 (4.3%)	0 (0%)	

WEDT, dual task water-based exercise group; WE, water-based exercise group; CG, control group; BMI, body mass index; WHR, waist-to-hip ratio; SM, Brazilian minimum wage; *Indicates a significant difference (p < 0.05).

**Table 2** shows the cognitive outcomes. The total MoCA score showed no main effects of time or group, and no group × time interaction (p > 0.05). Among MoCA domains, only orientation showed a main effect of time (p = 0.006), with no between-group differences or relevant interactions. The remaining cognitive measures (phonemic verbal fluency - letters F and M - and Trail Making Tests Parts A and B) showed no significant effects of time, group, or interaction (p > 0.05). In contrast, semantic verbal fluency (animals) showed a significant group × time interaction (p = 0.003), with an increase in the WEDT group, a decrease in the WE group, and no change in the CG. Effect sizes were predominantly small for the total MoCA score and its domains.

**Table 2:** Cognitive results in the intention-to-treat analysis (MoCA, verbal fluency, and trail making test).

Variable	Group	n	Pre-intervention			Post-intervention			Hedges’ g (CI 95%)	Group	Time	Group
			Mean	CI 95%		Mean	CI 95%					*
				Lower	Upper		Lower	Upper				Time
Total MoCA	WE	23	22.5	21.0	24.0	23.1	21.6	24.5	0 (-0.5; 0.6)	0.88	0.394	0.730
	WEDT	23	23.1	21.6	24.5	23.5	22.0	25.0	0.18 (-0.4; 0.8)			
	CG	21	23.0	21.6	24.4	22.9	21.4	24.4				
Visuospatial/ executive	WE	23	3.3	2.7	3.9	3.1	2.5	3.6	-0.4 (-1.0; 0.2)	0.869	0.188	0.075
	WEDT	23	3.0	2.4	3.6	3.5	3.1	4.0	0 (-0.6; 0.6)			
	CG	21	3.2	2.6	3.7	3.5	3.1	3.9				
Naming	WE	23	2.7	2.5	2.9	2.9	2.7	3.0	0.1 (-0.5; 0.7)	0.212	0.314	0.057
	WEDT	23	2.7	2.3	3.1	2.5	2.1	2.8	-0.5 (-1.1; 0)			
	CG	21	2.9	2.8	3.1	2.8	2.6	3.0				
Attention	WE	23	4.4	3.9	5.0	4.5	4.0	5.1	0 (-0.6; 0.6)	0.732	0.535	0.701
	WEDT	23	4.8	4.4	5.2	4.6	4.2	5.1	0.1 (-0.5; 0.7)			
	CG	21	4.6	3.9	5.3	4.5	3.8	5.2				
Language	WE	23	1.7	1.3	2.1	1.7	1.2	2.1	0 (-0.6; 0.6)	0.29	0.527	0.715
	WEDT	23	1.9	1.5	2.3	2.0	1.6	2.5	0.4 (-0.2; 1)			
	CG	21	1.4	1.1	1.8	1.6	1.2	2.1				
PVF (Letter F)	WE	23	12.1	10.0	14.1	12.3	10.5	14.0	0.3 (-0.3; 0.9)	0.638	0.432	0.167
	WEDT	23	11.8	9.7	13.9	13.7	11.5	16.0	-0.5 (-1.1; 0.1)			
	CG	21	11.7	9.3	14.0	10.9	8.1	13.7				
Abstraction	WE	23	1.1	0.8	1.5	1.3	1.0	1.6	-0.4 (-1.0; 0.2)	0.065	0.375	0.64
	WEDT	23	1.4	1.1	1.8	1.6	1.2	2.0	-0 (-0.6; 0.5)			
	CG	21	1.7	1.5	1.9	1.6	1.3	2.0				
Delayed recall	WE	23	2.8	2.2	3.4	3.5	3.0	3.9	0.6 (-0; 1.2)	0.473	0.546	0.073
	WEDT	23	3.1	2.5	3.7	2.9	2.3	3.5	0.1 (-0.5; 0.7)			
	CG	21	2.8	2.2	3.3	2.8	2.2	3.3				
Orientation	WE	23	5.8	5.6	6.0	5.6	5.3	5.8	0 (-0.6; 0.6)	0.888	0.006*	0.482
	WEDT	23	5.8	5.5	6.0	5.7	5.4	6.0	0.2 (-0.4; 0.7)			
	CG	21	5.9	5.8	6.1	5.6	5.2	5.9				
PVF (Letter M)	WE	23	12.1	10.2	14.1	12.1	10.0	14.3	0 (-0.6; 0.5)	0.451	0.782	0.968
	WEDT	23	13.6	11.6	15.6	13.9	11.7	16.1	0.3 (-0.3; -0.9)			
	CG	21	12.2	9.8	14.7	12.4	9.6	15.1				
SVF (Animals)	WE	23	16.6	14.3	19.0	14.8#	12.7	16.9	-0.4 (0.2; 1.0)	0.702	0.649	0.003*
	WEDT	23	15.8	14.1	17.6	18.0#	15.7	20.3	0.3 (-0.3; 0.9)			
	CG	21	16.2	12.9	19.5	16.6	14.3	18.9				
Trail A (s)	WE	23	55.0	45.8	64.2	60.1	44.8	75.4	0.5 (-0.1; 1.1)	0.071	0.321	0.277
	WEDT	23	56.4	46.3	66.6	63.1	46.1	80.1	0.6 (0; 1.2)			
	CG	21	47.5	41.0	54.0	45.4	38.6	52.1				
Trail B (s)	WE	23	155.6	128.7	182.5	148.2	110.3	186.1	0.1 (-0.5; 0.7)	0.74	0.275	0.754
	WEDT	23	174.7	138.7	210.7	150.5	111.5	189.5	0.1 (-0.5; 0.7)			
	CG	21	149.4	113.6	185.3	143.3	118.3	168.2				

WEDT, Water-based Exercise with Dual Task group; WE, Water-based Exercise group; CG, Control Group; MoCA, Montreal Cognitive Assessment; PVF, Phonemic Verbal Fluency; SVF, Semantic Verbal Fluency. *Indicates a significant difference (p < 0.05); #indicate differences over time. n indicates the number of participants randomized to each group; analyses were based on all available observations.

**Table 3** summarizes mobility, cardiorespiratory fitness, and neuromuscular outcomes in the intention-to-treat analysis. For mobility, the TUG showed no main effects of group or time, and no group × time interaction (p > 0.05). By contrast, the dual-task TUG (TUG-DT) showed a significant group × time interaction (p = 0.032), driven by improvements observed only in the CG. For the 6-min walk test, a between-group difference was observed (p = 0.020), with no main effect of time and no interaction (p > 0.05).

**Table 3:** Results for functional mobility, cardiorespiratory fitness, and neuromuscular outcomes analyzed by intention to treat.

Variable	Group	n	Pre-intervention			Post-intervention			Hedges’ g CI 95%	Group	Time	Group
			Mean	CI 95%		Mean	CI 95%					*
				Lower	Upper		Lower	Upper				Time
TUG (s)	WE	23	6.5	6.1	7.0	6.6	6.1	7.0	0.5 (0; 1.2)	0.124	0.883	0.189
	WEDT	23	6.6	6.1	7.1	6.8	6.3	7.3	0.9 (0.3; 1.5)			
	CG	21	6.3	6.0	6.7	6.1	5.7	6.4				
TUGdt (s)	WE	23	7.3	6.7	7.9	7.5	6.7	8.3	0.7 (0.1; 1.3)	0.159	0.691	0.032*
	WEDT	23	7.4	6.8	8.0	7.7	6.9	8.6	0.8 (0.2; 1.4)			
	CG	21	7.3	6.8	7.7	6.6#	6.1	7.1				
6MWT (m)	WE	23	493.0	466.0	520.0	506.2	480.1	532.4	-0.3 (-0.9; 0.3)	0.020*	0.154	0.974
	WEDT	23	453.5	422.8	484.1	464.7	432.0	497.2	-0.9 (-1.5; -0.2)			
	CG	21	510.5	472.1	548.8	527.3	487.8	566.9				
HGS (kg)	WE	23	25.1	20.8	29.5	30.5#	27.1	33.8	0.3 (-0.3; 0.9)	0.845	0.005*	0.037*
	WEDT	23	26.7	23.8	29.6	27.5	24.6	30.4	0 (-0.6; 0.5)			
	CG	21	27.0	22.9	31.2	27.9	24.0	31.8				
LLME (Rep)	WE	23	16.4	13.8	19.0	18.1	15.6	20.7	0.6 (0; 1.2)	0.154	0.113	0.519
	WEDT	23	14.4	13.4	15.5	15.1	13.5	16.6	-0.1 (-0.7; 0.5)			
	CG	21	15.2	13.2	17.2	15.4	13.6	17.1				
MVIC (Kgf)	WE	23	20.2	15.7	24.7	18.4	14.4	22.4	-0.1 (-0.7; 0.4)	0.511	0.640	0.014*
	WEDT	23	19.4	14.0	24.8	21.2	16.4	25.9	0.1 (-0.5; 0.7)			
	CG	21	13.2	10.1	16.3	20.0#	13.0	26.9				
RF (μV)	WE	23	87.4	65.5	109.3	114.8#	74.6	154.9	0.5 (-0.1; 1.1)	0.294	0.008*	0.028*
	WEDT	23	79.1	57.1	101.0	101.7#	79.7	123.8	0.2 ( -0.4; 0.7)			
	CG	21	79.4	62.8	96.0	75.8	61.2	90.5				
VL (μV)	WE	23	93.2	70.6	115.8	108.4	77.1	139.8	0 (-0.6; 0.6)	0.896	0.233	0.808
	WEDT	23	107.8	77.4	138.1	112.0	79.2	144.9	0(-0.6; 0.6)			
	CG	21	101.0	79.8	122.0	110.1	67.0	153.2				

TUG, Timed up Go; TUDdt, Timed up Go with dual task; 6MWT, 6-minute walk test; HGS, Handgrip Strength; LLME, Lower-limb Muscular Endurance; Rep, repetition; MVIC, Maximal Voluntary Isometric Contraction; RF, Rectus Femoris; VL, Vastus Lateralis; WEDT, Water-based Exercise with Dual Task group; WE, Water-based Exercise group; CG, Control Group. *indicates a significant difference (p < 0.05), #indicate differences over time. n indicates the number of participants randomized to each group; analyses were based on all available observations.

Regarding neuromuscular outcomes, handgrip strength showed a significant group × time interaction (p = 0.037), with increases observed only in the WE group, whereas the WEDT and control groups remained stable. Lower-limb muscular endurance showed no effects of time, group, or interaction (p > 0.05). For MVIC, a significant group × time interaction was observed (p = 0.014), characterized by an increase in the CG and maintenance in both intervention groups.

In terms of muscle activation, the rectus femoris showed a significant group × time interaction (p = 0.028), with increases in both intervention groups and no change in the control group, whereas the vastus lateralis showed no significant effects (p > 0.05). Effect sizes were predominantly small to moderate, with moderate effects particularly evident for lower-limb muscular endurance and rectus femoris activation in the WE group.

As a sensitivity analysis (**Supplementary Table S3**), the per-protocol (PP) analysis revealed some differences compared with the intention-to-treat (ITT) findings. There was a significant interaction for the total MoCA score, with improvement in the WE group and maintenance in the WEDT and CG. Specific MoCA domains, including naming, abstraction, and delayed recall, also showed significant interactions: the WEDT group improved in abstraction, the WE group improved in delayed recall, and the CG remained stable across most domains.

For physical outcomes, the PP analysis retained the significant group × time interaction for the TUG-DT (p = 0.005), with improvement observed only in the CG. In addition, aerobic capacity (6-min walk test) improved over time regardless of group.

For neuromuscular outcomes, the PP analysis indicated significant interactions for handgrip strength (increase in the WE group), MVIC (decrease in the WE group), and rectus femoris activation (increase in the WEDT group), whereas the remaining outcomes showed no significant changes. Overall, these supplementary findings underscore the importance of adherence. However, the primary interpretation remains based on the ITT analysis, because it preserves the benefits of the initial randomization.

## 4 Discussion

This study investigated whether adding a dual-task component to water-based exercise would provide incremental benefits in global cognition among older women. Our findings did not demonstrate the superiority of the dual-task group for the primary outcome. Overall, most cognitive and physical outcomes showed little change in the intervention groups, whereas some improvements were observed in the control group. Taken together, these results suggest that, in real-world program settings, adding a cognitive task to aquatic exercise may be insufficient to elicit additional cognitive benefits unless factors such as training dose and adherence are carefully considered.

Our findings are consistent with the existing literature, which reports mixed results for conventional aquatic interventions and dual-task aquatic programs on cognitive outcomes (Fedor et al., 2015; Sato et al., 2015; Kang et al., 2020; Campos et al., 2021; Farinha et al., 2021; Campbell et al., 2023; Häfele et al., 2023; Dunlap et al., 2024; Terra de Oliveira et al., 2024; Meekum et al., 2025). Potential explanations for the lack of significant effects include the limited sensitivity of the MoCA to detect subtle changes, particularly in samples with higher baseline scores (Lindvall et al., 2024). Moreover, the relatively preserved cognitive status of the participants may have reduced the potential for detectable improvement because of possible ceiling effects (Meekum et al., 2025).

The cognitive training dose may also have been insufficient to induce broader adaptations. Concurrent cognitive stimulation accounted for only 10 minutes of each session, and the combination of two weekly sessions with moderate adherence further reduced the cumulative exposure actually received. Based on the mean adherence observed in the WEDT group, this corresponded to approximately 2.5 hours of concurrent cognitive–motor training across the entire 14-week intervention. Such limited cumulative exposure may have attenuated the potential intervention effect and reduced the likelihood of detecting between-group differences in global cognition. Therefore, the findings should be interpreted as reflecting the effects of the intervention under the dose actually received rather than the full planned training dose. In addition, participant losses and the resulting reduction in the effective sample size may have limited the statistical power to detect differences in some secondary outcomes, increasing the possibility of type II error. Future studies should investigate whether longer or more frequent cognitive–motor exposure, more challenging tasks, and strategies to improve adherence produce broader cognitive benefits.

Notably, although we did not observe an overall benefit for global cognition, the dual-task group showed a specific improvement in semantic verbal fluency (animals). This finding suggests that certain executive processes may respond to targeted cognitive stimulation even over a relatively short period. Such improvements may be relevant for older women, as verbal fluency is linked to social participation and broader psychosocial/functional factors in older adults, which are key components of everyday communication and engagement (Francisco et al., 2022; Fernández et al., 2023). The improvement observed in semantic verbal fluency may be partially explained by the nature of the dual-task activities performed during the intervention. Several dual-task activities required semantic retrieval, verbal categorization, lexical access, and memory recall while participants simultaneously executed motor tasks. These repeated demands may have preferentially stimulated semantic memory and lexical retrieval processes, thereby promoting task-specific adaptations, which could explain the superior performance observed in semantic verbal fluency after training. This interpretation is consistent with the principle of training specificity, whereby cognitive domains that are repeatedly stimulated during intervention tend to demonstrate greater post-intervention gains.

Across physical performance outcomes, the WE group was not superior to the WEDT or CG for most measures, and effects were generally modest. We speculate that the dose received may have been insufficient to induce robust adaptations in mobility, cardiorespiratory fitness, and neuromuscular outcomes, particularly given the observed adherence, which likely limited the overall physiological stimulus.

Notably, the WE group showed an increase in handgrip strength, a widely used marker of overall health status that has been associated with morbidity and mortality risk in older populations (Soysal et al., 2021). However, this effect was not accompanied by consistent superiority across more specific physical and neuromuscular outcomes and therefore should be interpreted as a domain-specific signal rather than evidence of a broader performance advantage. Still, the finding is relevant because handgrip strength is commonly used in clinical and public health contexts and may reflect general neuromuscular status.

The improvements observed in the CG for TUG-DT and MVIC may reflect a combination of non-specific, contextual, and practice-related factors rather than a true intervention effect. Study participation itself may have triggered indirect behavioral changes (research participation/Hawthorne effects), such as increased engagement in daily activities or greater attention to routine behaviors, even in the absence of a structured program (McCambridge et al., 2014). In addition, unmeasured changes in physical activity outside the study could have contributed to these findings, as objective monitoring of out-of-program activity was not performed. Learning or practice effects associated with repeated exposure to the assessment procedures may also have contributed, particularly because no formal familiarization sessions were conducted. This possibility may be especially relevant for MVIC, for which greater familiarity with the testing procedure and maximal effort requirements at post-intervention could have contributed to the substantial increase observed in the control group. Taken together, these considerations suggest that the unexpected control-group changes should be interpreted cautiously and highlight the importance of familiarization procedures and objectively monitoring physical activity levels in future trials.

Adherence to the interventions was moderate in the ITT analysis (55.2% ± 10.36% in the WEDT group and 65.7% ± 9.89% in the WE group), indicating that the dose received was lower than planned for a substantial proportion of participants. In addition, dropouts occurred predominantly due to intercurrent health issues (n = 13), suggesting that, in programs targeting older women, health-related events are expected and may attenuate exercise responses. In this context, the trial also provides relevant information on the feasibility and effectiveness of the intervention under conditions that approximate real-world settings.

Findings from the PP analysis, restricted to participants with ≥ 70% attendance, further support that adherence may influence response patterns for some outcomes, although these estimates should be interpreted as sensitivity analyses given the potential for selection bias. For future studies and practical implementation, strategies to mitigate attrition and sustain participation may be necessary to ensure a sufficient training dose and maximize potential benefits, for example, offering make-up sessions after health-related interruptions and providing greater scheduling flexibility. While such strategies can be challenging to operationalize in clinical trials, they may be feasible in community-based programs.

Several limitations should be considered. First, intervention fidelity could not be fully established. Although exercise intensity was prescribed using target Borg RPE ranges, individual RPE values were not systematically recorded during or after the sessions. Therefore, we could not verify whether participants consistently achieved and maintained the intended training intensity, and the actual physiological dose remains uncertain. In addition, physical activity performed outside the intervention was not objectively monitored. Consequently, unmeasured changes in habitual activity may have contributed to some findings, particularly the unexpected improvement in TUG-DT observed in the control group.

Second, several limitations related to assessment and statistical analysis should be acknowledged. Formal familiarization sessions were not conducted for the cognitive and functional assessments; therefore, repeated exposure may have produced practice effects, particularly for timed and dual-task measures, although all groups completed the same assessment schedule. In addition, because multiple secondary outcomes were evaluated, isolated statistically significant findings should be interpreted cautiously due to the increased probability of type I error.

Among the main strengths, the three-arm randomized controlled trial design is noteworthy, as it allowed us to isolate the potential incremental effect of adding dual-task demands beyond conventional aquatic exercise. The study also included a comprehensive battery of outcomes, encompassing global cognition, specific measures related to executive function/attention, and cardiorespiratory and neuromuscular components, thereby providing an integrated view of potential benefits and trade-offs of the protocol. In addition, a methodological contribution of this trial was the periodization of the cognitive component within the dual-task intervention, with a planned progression across weeks based on MoCA domains, facilitating replication in community-based programs. Although dual-task approaches have been increasingly applied, protocols describing cognitive progression in a clear and structured manner remain ​​scarce in the literature, which limits reproducibility.

Accordingly, we conclude that, in older women, a 14-week (2 sessions/week) water-based exercise program with dual-task demands did not demonstrate a consistent additional benefit for global cognition compared with conventional water-based exercise, and effects on physical performance were generally modest. For implementation in community-based programs, these findings suggest that adding a dual-task component may be insufficient without high adherence. Therefore, the absence of consistent additional cognitive benefits should be interpreted in light of the moderate adherence achieved, which reduced the planned intervention dose. Moreover, when improving physical performance is the primary goal, it may be preferable to alternate sessions with a motor emphasis and a cognitive–motor emphasis and/or to deliver dedicated sessions focused on cognition. Future trials should test higher training doses/frequencies, strategies to maximize adherence, and familiarization and monitoring procedures (including objective assessment of physical activity outside the protocol) to clarify under which conditions dual-task aquatic exercise can produce clinically meaningful gains.

## Data Availability

The raw data supporting the conclusions of this article will be made available by the authors, without undue reservation.
