# Supplementary material for: Added effects of dual-task water-based exercise on global cognition and physical performance in older women: a three-arm randomized controlled trial
Source: Front Physiol. 2026 Sep 25;17:1866915. doi: 10.3389/fphys.2026.1866915 (PMC13649429; doi:10.3389/fphys.2026.1866915)
Supplement: Supplementary file 1 [file DataSheet1.pdf]

Supplementary material

Table 1. Session plan template applied to both groups

Time (minute by minute)	Lower limb exercise	Upper limb exercise	Intensity (IEP) Mesocycle 1	Intensity (IEP) Mesocycle 2	Intensity (IEP) Mesocycle 3	Intensity (IEP) Mesocycle 4	Intensity (IEP) Mesocycle 5
0 - 7	Mobilization of large muscle groups		9	9	9	9	9
8	Stationary Run	Alternate Hug	13	15	15	16	15*
9	Diagonal Slide	Hug	13	15	17	19	17
10	Diagonal Slide	Alternate Hug	13*	15*	15*	13*	19
11	Stationary Run	Alternate Elbow Flex/Ext	13	15	15	16	15*
12	Lateral Slide	Simultaneous Elbow Flex/Ext	13	15	17	19	17
13	Lateral Slide	Alternate Elbow Flex/Ext	13*	15*	15*	13*	19
14	Stationary Run	Alternate Shoulder Flex/Ext	13	15	15	16	15*
15	Front Kick	Simultaneous Shoulder Flex/Ext	13	15	17	19	17
16	Front Kick	Alternate Shoulder Flex/Ext	13*	15*	15*	13*	19
17	Front Slide	Chest Arm	13	15	15	16	15*
18	Stationary Run	Arm accompanies movement	13	15	17	19	17
19	Front Slide	Chest Arm	13*	15*	15*	13*	19
20	Lateral Slide	External Shoulder Rotation	13	15	15	16	15*
21	Front Kick	Alternate Front Push	13	15	17	19	17
22	Lateral Slide	Alternate Front Push	13*	15*	15*	13*	19
23	Stationary Run	Shoulder Adduction and Abduction	13	15	15	16	15*
24	Posterior Run	Shoulder Flex/Ext Vertical	13	15	17	19	17
25	Stationary Run	Adduction and Abduction and Shoulder Abduction	13*	15*	15*	13*	19
26	Stationary Run	Hug Alternate	13	15	15	16	15*

27	Diagonal Slide	Hug	13	15	17	19	17
28	Diagonal Slide	Hug Alternate	13*	15*	15*	13*	19
29	Stationary Run	Flex/Ext Elbow Alternate (palm down)	13	15	15	16	15*
30	Lateral Slide	Flex/Ext Elbow Simultaneous (palm down)	13	15	17	19	17
31	Lateral Slide	Flex/Ext Elbow Alternate (palm down)	13*	15*	15*	13*	19
32	Front Slide	Chest Arm	13	15	15	16	15*
33	Stationary Run	Arm follows the movement	13	15	17	19	17
34	Front Slide	Chest Arm	13*	15*	15*	13*	19
35	Lateral Slide	External Shoulder Rotation	13	15	15	16	15*
36	Front Kick	Push down Alternate	13	15	17	19	17
37	Lateral Slide	Push sideways Alternate	13*	15*	15*	13*	19
39 - 45	Stretching the main muscle groups exercised in the session		9	9	9	9	9
**IEP = Perceived effort index (Borg 6-20). * Indicates when the cognitive exercises were performed in the dual-task aquatic training group (WEDT).**							

Table 1. Structure of the periodization of the intervention groups

		MESOCYCLE 1<br>1-5 Weeks		MESOCYCLE 2<br>6-10 Weeks		MESOCYCLE 3<br>11-14 Weeks	
		**STRUCTURE**	**RPE**	**STRUCTURE**	**RPE**	**STRUCTURE**	**RPE**
**WE**		30 min	13	30 min	15	30 min	17
**WEDT**	10x	2 min motor activity<br>1 min dual task (motor+cognitive)	13	2 min motor activity<br>1 min dual task (motor+cognitive)	15	2 min motor activity<br>1 min dual task (motor+cognitive)	17
	Progression of cognitive domains						
	Working memory	During the warm-up, the following sequence of words was said: orange, ball, dog, stone, lion (repeated 3 times). After 5 minutes, the instructor asked: What were the words said?		During the warm-up, the following sequence of numbers and colors was said: red 3, blue 7, green 2 (repeated 3 times). After 5 minutes, the instructor asked: What were the colors said? What were the odd numbers? What was the color that started with the letter B?		During the warm-up, the following sequence of numbers and colors was recited: pink 78, brown 18, gray 95, green 17. After 5 minutes, the teacher asked: What was the sequence of numbers and colors in direct order? And in reverse order? What were the even numbers?	
	Language	The participants were instructed to say as many words as possible that began with the letter "P" in one minute. Proper nouns, cities, and verbs were not allowed. Example: pirate, pink, poor.		The participants were instructed to say as many words as possible containing the syllable "PA" in 1 minute. Proper nouns, cities, and verbs were not allowed. Example: package, parent, party.		The instructor would say the word HAPPY, for example, and the participants would have to say words that begin with the last letter of the word. Example: yes, young, you.	
	Attention	The instructor would say a sequence of random letters, and every time the students heard, for example, the letter D, they were to clap.		The instructor would say a sequence of numbers, and the participants would then repeat the sequence of numbers in direct order.<br>For example: 3-5-4-0-1.		The instructor would say a sequence of numbers, for example 358, and the participants would respond in reverse order, for example, 853.	

	Naming	The instructor showed a picture of an animal (for example, an alligator) and asked: What kind of animal is this? What is its habitat? What does it eat?	The instructor showed two pictures of animals (for example, a cat and a whale) and asked: Who are these animals? Which one is domestic? What are the characteristics of their fur? Where do they live? Which one is bigger? Does anyone know a breed of cat? Does anyone know a species of whale?	The instructor would show animals (for example, horse, cat, whale) and ask: Which animals begin with the letter C? Which animal names end with a vowel? Which animals are mammals? Which animal lives in the sea?
	Orientation	The instructor asked questions related to the time and place they were in. For example: What month is it? How many days are in this month? What season is it? What area of the city do you live in? What neighborhood do you live in? What neighborhood are we in?	The instructor asked the following questions: What squares and parks in Porto Alegre* do you know? Where is Farroupilha Park located? What neighborhood is it in? Where is Marinha Park located? What neighborhood is it in? Which neighborhoods in Porto Alegre are adjacent to the Jardim Botânico# neighborhood?	The instructor would ask the following questions: In which state of the country are we? What is our neighboring state? Do you know the names of the capitals of the states that make up the Southern region of Brazil? The instructor would say a capital (for example, Salvador) and ask which state it belonged to.
	File memory	The instructor asked the participants to name household utensils they used in the past for 1 minute.	The instructor asked the volunteers about daily life in cities and in the countryside during their childhood. For example, what means of transportation did they use?	The instructor asked them to name their high school or college classmates. Then she asked if the volunteers still kept in touch with those classmates and asked them to share a memory of that relationship.
	Executive function	The instructor instructed them to say a short sentence with one word. For example, love.	The instructor instructed them to say a single sentence with more words. For example, friendship, affection, and love.	The instructor presented a problem, and the participants had to think of solutions. For example: You are lost in a place, what would you do to get out?

Note: RPE, rating of perceived exertion (Borg 6-20); WE, water-based exercise group; WEDT, water-based exercise with dual-task group. *city where the participants lived; #neighborhood where the project classes took place.

Table 2. Results of the MoCA domains, verbal and semantic fluency, and trails A and B tests analyzed per protocol.

Variable	Group	n	Pre-intervention			Post- intervention			Hedges' g CI 95%	Group	Time	Group * Time
			Mean	CI 95%		Mean	CI 95%					
				Lower	Upper		Lower	Upper				
Total MoCA	WE	14	21.5	19.8	23.5	23.6#	21.7	25.4	0.2 (-0.4;0.8)	0.527	**0.001***	**0.041***
	WEDT	12	23.5	21.8	25.2	24.1	22.4	25.8	0.4 (-0.2;1)			
	CG	16	22.5	21.1	23.9	22.9	21.4	24.4				
visuospatial/executive	WE	14	3.1	2.4	3.7	3.1	2.5	3.7	-0.4 (-99;0.2)	0.827	**0.038***	0.305
	WEDT	12	3.1	2.3	3.9	3.6	3	4.2	0.1 (-0.5;0.7)			
	CG	16	2.9	2.4	3.5	3.5	3.1	3.9				
Naming	WE	14	2.7	2.5	3	2.9#	2.8	3.1	0.3 (-0.3;0.9)	0.497	0.976	**0.041***
	WEDT	12	2.7	2.2	3.2	2.6	2.2	3	-0.3 (-0.9;0.3)			
	CG	16	2.9	2.8	3.1	2.8	2.6	3				
Attention	WE	14	3.9	3.3	4.6	4.4	3.6	5.1	-0.1 (-0.7;0.5)	0.143	0.868	0.075
	WEDT	12	5.1	4.7	5.5	4.7	4.1	5.2	0.1 (-0.4;0.7)			
	CG	16	4.4	3.7	5.2	4.5	3.8	5.2				
Language	WE	14	1.4	1	1.9	1.6	1.1	2.1	-0.1 (-0.7;0.5)	**0.014***	0.522	0.91
	WEDT	12	2.2	1.8	2.6	2.2	1.7	2.6	0.6 (0;1.2)			
	CG	16	1.5	1.2	1.9	1.6	1.2	2.1				
PVF (Letter F)	WE	14	10.5	8.4	12.6	11.9	9.7	14.2	0.2 (-0.4;0.8)	0.132	0.285	0.059
	WEDT	12	12.8	10.4	15.2	14.7	12.5	16.8	0.8 (0.2;1.4)			
	CG	16	12.1	9.6	14.7	10.9	8.1	13.7				
Abstraction	WE	14	1.8	1.5	2.0	1.6#	1.3	2	0 (-0.6-0.6)	**0.007***	**0.006***	**0.016***
	WEDT	12	1.3	0.9	1.8	1.7#	1.3	2	0 (-0.5;0.6)			
	CG	16	1.8	1.5	2	1.6	1.3	2				
Delayed recall	WE	14	3.1	2.4	3.7	3.9#	3.3	4.4	0.9 (0.3;1.5)	**0.034***	0.176	**0.012***
	WEDT	12	3.3	2.7	3.8	3	2.3	3.7	0.2 (-0.4;0.9)			
	CG	16	2.6	2	3.1	2.8	2.2	3.3				

Orientation	WE	14	5.9	5.7	6	5.6	5.4	5.9	0.1 (-0.5;0.7)			
	WEDT	12	5.7	5.4	5.9	5.8	5.6	6	0.4 (-0.2;1)	<0.999	0.084	**0.039***
	CG	16	5.9	5.8	6.1	5.6#	5.2	5.9				
PVF (Letter M)	WE	14	11.1	9.2	13.1	11.5	8.8	14.1	-0.2 (-0.8;0.4)			
	WEDT	12	15.1	13.2	17	14.8	12.4	17.3	0.5 (-0.1;1.1)	**0.029***	0.917	0.935
	CG	16	12.3	9.5	15	12.4	9.6	15.1				
SVF (Animals)	WE	14	16.1	13.1	19.2	14.8	11.9	17.6	-0.3 (-0.9;0.2)			
	WEDT	12	16.8	14.7	18.8	18.9	16.3	21.5	0.5 (-0.1-1.1)	0.372	0.472	0.06
	CG	16	16	12.5	19.5	16.6	14.3	18.9				
Trail A (s)	WE	14	51.6	43.4	59.8	56	40.6	71.4	0.4 (-0.1;1)			
	WEDT	12	56.9	43.8	70.1	62.7	42	83.3	0.6 (0;1.2)	0.249	0.550	0.318
	CG	16	48.9	42	55.91	45.4	38.6	52.1				
Trail B (s)	WE	14	165.2	127.5	202.8	154.5	103.6	205.4	0.1 (-0.4;0.7)			
	WEDT	12	166.8	124.7	208.8	139.1	91.5	186.7	-0.1 (-0.6;0.5)	0.889	0.234	0.840
	CG	16	155.6	115.8	195.3	143.3	118.3	168.2				

Note: WEDT, Water-based Exercise with Dual Task group; WE, Water-based Exercise group; CG, Control Group; MoCA, Montreal Cognitive Assessment; PVF (Letter F), Phonemic Verbal Fluency taken from the MoCA; PVF (Letter M), Phonemic Verbal Fluency; SVF, Semantic Verbal Fluency.* indicates a significant difference ($p < 0.05$); # indicate differences over time.

Table 3. Results for functional mobility, cardiorespiratory fitness, and neuromuscular outcomes analyzed per protocol.

Variable	Group	n	Pre-intervention			Post- intervention			Hedges' g CI 95%	Group	Time	Group * Time
			Mean	CI 95%		Mean	CI 95%					
				Lower	Upper		Lower	Upper				
TUG (s)	WE	14	6.6	5.9	7.2	6.6	6.2	7.1	0.7 (0.1;1.3)	0.27	0.524	0.099
	WEDT	12	6.7	6.0	7.3	6.8	6.2	7.3	0.8 (0.2;1.4)			
	CG	16	6.5	6.2	6.7	6.1	5.7	6.4				
TUGdt (s)	WE	14	7.5	6.7	8.4	7.8	6.9	8.7	0.8 (0.2;1.4)	0.243	0.499	**0.005***
	WEDT	12	7.3	6.5	8.1	7.6	6.6	8.6	0.7 (0.1;1.4)			
	CG	16	7.5	7.1	7.8	6.6#	6.1	7.1				
6MWT (mts)	WE	14	485.9	458.7	513.0	502.6	468.6	536.6	-0.3 (-0.9;0.3)	0.057	**0.018***	0.849
	WEDT	12	444.0	406.4	481.7	463.3	425.6	501.0	-0.8 (-1.5; -0.2)			
	CG	16	496.2	459.2	533.1	527.3	487.8	566.9				
HGS (kg)	WE	14	26.4	23.2	29.7	30.1#	25.6	34.5	0.3 (-0.3;0.9)	0.881	**0.003***	**0.029***
	WEDT	12	27.3	23.6	30.9	27.8	24.5	31.1	0 (-0.6;0.6)			
	CG	16	27.3	23.1	31.5	27.9	24.0	31.8				
LLME (Rep)	WE	14	17.2	14.4	20.0	18.0	14.6	21.4	0.4 (-0.2;1)	0.381	0.209	0.757
	WEDT	12	15.0	13.6	16.4	15.9	14.4	17.4	0.1 (-0.5;0.7)			
	CG	16	15.2	13.2	17.2	15.4	13.6	17.1				
MVIC (Kgf)	WE	14	21.3	15.5	27.1	18.8#	13.6	23.9	-0.2 (-0.8;0.4)	0.758	0.233	**0.018***
	WEDT	12	20.6	12.8	28.4	22.4	15.6	29.1	0.1 (-0.5;0.7)			
	CG	16	15.6	11.1	20.1	20.8	13.4	28.2				
RF (μV)	WE	14	94.7	62.0	127.0	122.2	62.2	182.2	0.5 (-0.0;1.1)	0.352	**0.035***	**0.012***
	WEDT	12	84.6	57.1	112.0	111.8#	81.4	142.3	0.9 (0.3;1.5)			
	CG	16	84.1	60.4	107.8	75.8	61.7	90.5				

	WE	14	91.2	62.8	119.6	112.9	71.4	154.5	0 (-0.5;0.6)			
VL (μV)	WEDT	12	110.3	73.3	147.2	131.9	90.5	173.3	0.3 (-0.3;0.9)	0.734	0.056	0.579
	CG	16	104.6	74.2	135.0	108.9	62.0	155.9				

Note: TUG, Timed up Go; TUDdt, Timed up Go with dual task; 6MWT, 6-minute walk test; HGS, Handgrip Strength; LLME, Lower-limb Muscular Endurance; MVIC, Maximal Voluntary Isometric Contraction; RF, Rectus Femoris; VL, Vastus Lateralis; WEDT, Water-based Exercise with Dual Task group; WE, Water-based Exercise group; CG, Control Group.* indicates a significant difference ($p < 0.05$); # indicate differences over time.
